# Impact of a sensory stimulation program conducted by family members on the consciousness and pain levels of ICU patients: A mixed method study

**DOI:** 10.3389/fmed.2022.931304

**Published:** 2022-09-20

**Authors:** Mohammad Adineh, Nasrin Elahi, Shahram Molavynejad, Simin Jahani, Mohsen Savaie

**Affiliations:** ^1^Student Research Committee, School of Nursing and Midwifery, Ahvaz Jundishapur University of Medical Sciences, Ahvaz, Iran; ^2^Nursing Care Research Center in Chronic Diseases, School of Nursing and Midwifery, Ahvaz Jundishapur University of Medical Sciences, Ahvaz, Iran; ^3^Department of Anesthesiology and Intensive Care, School of Medicine, Ahvaz Jundishapur University of Medical Sciences, Ahvaz, Iran

**Keywords:** brain injury, intensive care unit (ICU), sensory stimulation program, family members, Glasgow coma scale (GCS), behavioral pain scale (BPS), mixed method study

## Abstract

**Background:**

The results of several studies show the different effects of a balanced sensory stimulation program (SSP) on patients with brain injury admitted to the intensive care unit (ICU), but these effects have been less studied based on mixed and comprehensive methods.

**Method:**

This mixed-method study involved 66 patients with brain injury admitted to the ICU who were allocated into intervention (*n* = 33) and control (*n* = 33) groups using random stratified sampling. Patients in the intervention group received a sensory stimulation program from family members for 1 h daily during ICU hospitalization, while the control group received only routine care. Patients' level of consciousness and pain intensity were measured immediately before and after the intervention using Glasgow Coma Scale (GCS) and Behavioral Pain Scale (BPS), respectively. In-depth unstructured interviews were conducted with the patients in the intervention group 3 months after discharge from the ICU. These interviews were analyzed following Graneheim and Lundman (2004) conventional content analysis method.

**Results:**

A significant difference was found between the study groups in terms of the mean difference of GCS (*P* =0.001) and BPS score (*P* = 0.001) before and after intervention. Patients in the intervention group had a higher mean GCS and a lower mean BPS than did patients in the control group. The main themes extracted from the qualitative analysis confirmed the results obtained from the quantitative phase of the study.

**Conclusion:**

The combination of the quantitative and qualitative findings suggested that amidst the many hardships and sufferings brain injury patients go through in the ICU, a sensory stimulation program offered by family members may have many benefits such as increased level of consciousness and reduced pain for these patients. Therefore, it is necessary to formulate a framework for this program and provide the needed facilities in order to benefit more from the capacity of such programs for ICU patients.

## Introduction

Patients with moderate to severe brain injury are typically admitted to the intensive care unit (ICU) (1). They are exposed to sensory overload or deprivation for reasons such as damage to the structure and function of the brain, being in an isolated and unfamiliar environment, long-term use of the ventilator, failure to receive appropriate and balanced sensory stimuli for the five main senses, excessive intake of sedatives, and excessive and meaningless sensory stimuli such as noise made by the personnel and devices in the ward along with many painful, invasive procedures (2). Rapid, accurate, and scientific primary care and treatment for patients with brain injury in the emergency room and ICU significantly accelerates the recovery process. It prevents complications and permanent mental and physical disabilities in these patients (3). Prevention of sensory deprivation or overload is one of the important nursing cares for patients with brain injury in the ICU (1) since it can be associated with many negative consequences after ICU discharge, namely reticular activating system (RAS) suppression, cortical dysfunction, modification and plasticity, and even long-term cognitive impairment (4, 5).

In addition to different pharmacological and nonpharmacological methods used to prevent sensory deprivation or overload and its consequences in patients with brain injury in the ICU, using novel, low-cost, and effective nonpharmacological methods is among the priorities of evidence-based treatment and care (6). One of the nonpharmacological methods to improve the nervous system's function and increase consciousness is receiving balanced sensory stimulation (7). Several studies have shown the different effects of balanced sensory stimulation on patients with brain injury admitted to the ICU. For example, Li et al.'s study (8) showed that balanced sensory stimulation increases the consciousness level and arousal of patients with brain injury admitted to the ICU. In Another study, Jagan et al. (9) also found that massage and touch therapy interventions can positively affect the patients' consciousness and pain levels in the ICU. In the meantime, several other studies have indicated that sensory stimulation has better consequences for the patient if performed by people such as family members who are familiar to the patient (10–15). Adinehvand et al. (10) for instance, concluded that brain injury patients admitted to the ICU who receive sensory stimulation from family members have better outcomes in terms of their consciousness level and hemodynamic stability than those receiving sensory stimulation from nurses. In addition, according to the results of Khojeh et al. (16), auditory stimulation by family members' voice reduces pain intensity in patients admitted to the intensive care unit. The results and suggestions of these studies indicate the need for more comprehensive studies in this area. In fact, there is paucity of comprehensive studies dealing with not only the use of sensory stimulation program (SSP) (which involves stimulating all the patient's senses) as their intervention but also explaining the patients' experiences in order to achieve a deeper and more complete understanding of the subject under study and identify points that cannot be examined relying solely on quantitative studies.

Unlike patients in other units of hospital, ICU patients are often in a coma during their stay. They do not have the opportunity to express their experiences and preferences to the ICU healthcare personnel and researchers. Nevertheless, one of the important components of assessing the quality of care provided by healthcare personnel to patients admitted to the ICU is these patients' description of their experiences, which can be a suitable clinical guide for the healthcare personnel to provide quality care and for researchers to interpret research results with more rigor (17). Knowing the patients' experiences enables healthcare personnel (especially nurses) to be aware of the issues that the patients struggle with during their stay in the ICU. Armed with this knowledge, nurses can plan and implement care approaches that best meet the needs of these patients (18). Quantitative studies alone usually cannot provide such insight and knowledge to healthcare personnel and researchers. However, using a mixed-method study by combining and comparing quantitative and qualitative results can lead to more and better insights and knowledge in relation to the subject under study (19). In fact, in mixed-method studies, quantitative data ensures generalizability while qualitative data provides detailed information about the very context and situation in which the study takes place, and this provides the basis for not only a more comprehensive interpretation but also a deeper insight into the subject under consideration (20).

The literature tells us little about the effect of conducting SSP by family members on brain injury patients hospitalized in ICU, and there is an increasing need for more comprehensive evidence on this subject. Therefore, the present study was designed with a mixed-method approach in which the results of the quantitative phase are complemented with those of the qualitative phase involving interviews with patients describing their experiences about receiving SSP by family members. This will serve as the basis for extending the previous evidence and achieving a deeper understanding about this subject.

## Materials and methods

### Design

The present study is a mixed-method study using a “convergent parallel” approach with equal weight, conducted from June 2021 to march 2022 in the ICUs of Golestan Hospital affiliated to Ahvaz Jundishapur University of Medical Sciences, Ahvaz, Iran. The convergent parallel method is one of the common structures in mixed-method studies. Convergent parallel design is usually used when the researcher aims to directly compare quantitative results with qualitative findings, validate quantitative results using qualitative findings, or present those results in more detail (21). In this method, data collection and analysis of quantitative and qualitative phases of the study are performed independently but simultaneously (22).

The advantage and goal of the convergent parallel approach is that the study's qualitative phase highlights elements that may not be identified in quantitative data collection (23). In this study, the quantitative phase was performed as a two-group single-blind clinical trial to determine the effect of SSP performed by family members on the consciousness level and pain intensity of patients with brain injury admitted to the ICU. The qualitative phase was a conventional content analysis study to explain patients' experiences with ICU admission and receiving sensory stimulation from family members. Patients who were members of the intervention group and were discharged from the ICU were followed up simultaneously with the quantitative phase. They were invited for an interview at least 3 months after discharge if they were willing to participate.

### Participant recruitment

In the clinical trial phase of the study, 66 patients were selected based on the inclusion criteria and were then allocated randomly into two groups of intervention (*n* = 33) and control (*n* = 33) using random stratified sampling method. First, categories were formed based on age group with an interval of 10 years (18–27, 28–37, 38–47, 48–57, and 58–67 years) and then in each category, a random sequence was created using a table of random numbers. The sample size was calculated based on the results of Abbasi et al.'s study (13) in which the means of Glasgow coma scale (GCS) scores in their groups were 6.8 ± 1.4 and 7.8 ± 0.70. Accordingly, with a confidence level of 0.95 and a power of 0.90, we concluded that 30 patients were needed for each study group. Assuming a 10% attrition rate, the final sample size was set 33 people for each group.

Patients eligible for this phase of study were those diagnosed with acute brain injury, obtaining a GCS between 6 and 12 on admission, receiving similar medications to relieve pain, being intubated and under ventilator, receiving no prescribed neuromuscular blocking agents, being aged between 18 and 67 years, having pupillary reflexes, have not passed more than 2 days their admitted to ICU, and having no history of alcohol and substance abuse. Moreover, patients were excluded from the study if they were transferred to other hospitals during the study, entered persistent vegetative state, had hemodynamic instability, or were on continuous administration of neuromuscular blocking agents during the study.

In the qualitative phase of the study, 12 patients in the intervention group from the quantitative phase were selected to be interviewed using the purposive sampling method. To maximize data diversity, the participants were selected from patients with different diagnoses, sexes, ages, and lengths of stay in different ICUs. In this phase of the study, inclusion criteria were: membership in the intervention group in the quantitative phase, having a full consciousness level, and willingness to share experiences.

### Intervention

In this study, patients in the intervention group received (in addition to routine care) SSP by a family member (father, mother, sister, brother, or child) for 1 h a day, from 4 to 5 pm during their ICU stay. This family member did not replace during the study. In routine care scenario, patients do not receive any specific sensory stimulation program to stimulate all their senses, and the ICU patients' families are usually allowed to visit their patients sporadically only for a short and limited time. The SSP was performed as follows: First, consciousness stimulation was performed by saying the patient's name as well as the time and place near the patient's ear thrice per hour. Then, the patient's favorite music or family members' voices were played for 10 min for auditory stimulation. Next, for visual stimulation, family photos, videos, and beautiful pictures of interest were kept in front of the patient's eyes for 10 min. Then, aromatic stimuli and aromas to which the patient was more habituated were given for 10 s before the patient's nose for olfactory stimulation. In the next stage, tactile stimulation was performed once an hour by hand pressure, massage, and rubbing of the limb skin, first on one side of the body and then on the other side. Motor stimulation was performed in the last stage by moving the joints of the limbs, wrists, hips and shoulders in the normal range of motion by flexion and extension and alternatively moving the arms and legs up and down, 15 times per hour for each limb.

### Data collection

During the first 7 days of admission in ICU, immediately before and after each intervention, the patients' consciousness level and pain intensity were measured and recorded using GCS and behavioral pain scale (BPS), respectively. The nurses, who evaluated the GCS and BPS scores, were blinded to patient group allocation. BPS and GCS scales are the world standards for measuring the consciousness level and pain, especially in patients with brain injury admitted to the ICU (24, 25). Adinehvand et al. (10) obtained a correlation coefficient of 0.86 by the test-retest method for the GCS scale in Iran. Also, Arbabi et al. (26) obtained a Cronbach alpha coefficient of 0.84 for the BPS scale.

To explain the patients' experiences in the qualitative phase, data were collected through unstructured in-depth, face-to-face interviews with open-ended questions and audio recordings with patient permission. Depending on the participants' tolerance and willingness, the interviews lasted from 25 to 60 min. The interviews continued until data saturation. In qualitative studies, data collection (interviewing) continues until no new code or sub-theme is extracted from the interviewees' statements. Data saturation is a guide to decide on the sufficient number of interviews. In this way, if no new information (absence of new codes and sub-themes) is added at the time of data collection and the researchers come up with only cases that confirm the previous content at the time of collecting and updating the extracted information, they will end the sampling procedure (27). In this study, data saturation was achieved after interviewing the ninth participant, but the interviews were conducted with three more participants to ensure data saturation. Two patients were re-interviewed due to the inadequate content of the first interview. Therefore, a total of 14 interviews were conducted with 12 participants.

### Methodological rigor and trustworthiness

In this study trustworthiness was ensured based on Lincoln and Guba's criteria, namely Credibility, Dependability, Confirmability, and Transferability (28). To enhance credibility, the researchers were continuously involved in the process of implementing the study and allocated enough time to conduct the study and analyze the data. Moreover, all extracted data were reviewed and validated by the research team (peer check). The collected and analyzed data were also presented to the participants and they were asked if the narrative is accurate and a true reflection of their experience (member check). To enhance dependability, some of the interviews were provided to other researchers familiar qualitative research to check if they can also reach the same results and themes. To enhance confirmability, some of the interviews along with the codes, sub themes and extracted main themes were given to two qualitative analysis experts, outside the research team, in order to check the coding process in terms of accuracy. Finally, the codes and themes that needed serious modification were re-examined. To enhance transferability, an attempt was made to describe comprehensively the research context, participants, sampling method, and the time and place of data collection.

### Data analysis

Quantitative data analysis was conducted using SPSS version 22 and descriptive and analytical statistical tests, including independent-sample *t*-test (to compare the mean of continuous variables in two group), repeated-measures ANOVA test (to examine the changes of the GCS and BPS scores during consecutive measurement times) and Chi-square test (to compare nonparametric variables in two groups). Qualitative data were also analyzed in four stages based on Graneheim and Lundman (29) as follows: the interviews were first conducted and reviewed several times to better understand the entire content. The semantic units were then extracted and classified as compact units. In the next step, the compact units were summarized and categorized into sub-categories, and a suitable label for each was selected. Then, the sub-categories were arranged into categories based on their similarities and differences. An appropriate title was finally selected that could cover the resulting categories. In this study, data management was performed by MAXQDA software. Finally, to integrate the data, a comparative analysis was performed to identify similarities and differences between the main themes of the qualitative data and descriptive-analytical statistics of the quantitative data.

## Results

### Quantitative findings

**Figure 1:**
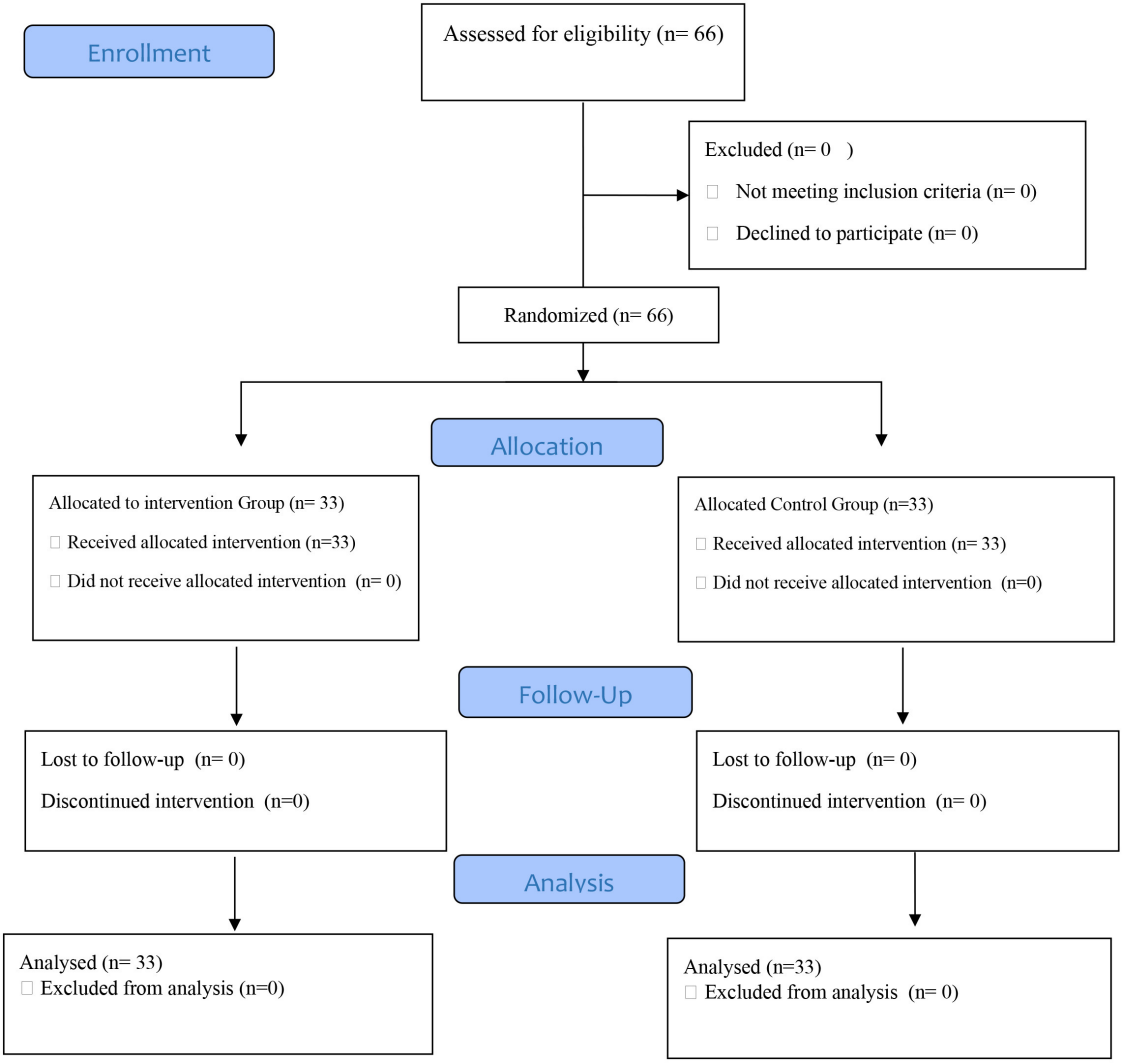
The Consort flowchart of patients participating in the study.

This mixed-method study involved 66 patients with brain injury admitted to the ICU who were divided into intervention and control groups using random stratified sampling (Figure 1). The mean age of participants was 36.18 ± 13.92 in the intervention group and 37.21 ± 13.98 in the control group and the mean Initial GCS of participants was 6.93 ± 0.7881 in the intervention group and 6.93 ± 0.74 in the control group. In the present study, 49 (74.2%) participants were male and 17 (25.8%) female. In terms of hospitalization diagnosis, most cases included 18 people (27.3%) Intracerebral Hemorrhages (ICHs) and in terms of the cause of brain injury, most cases (44 people = 66.7%) were accidents. The results showed no significant difference between the intervention and control groups regarding demographic and contextual variables. The more details are given in Table 1.

**Table 1:** Demographic characteristics of the ICU patients (*N* = 66)^a^.

**Variable**		**Group**		**Total**	***P*-value**
		**Intervention (*N* = 33)**	**Control (*N* = 33)**		
Age (year)	mean (SD)	36.18 (13.92)	37.21 (13.98)	36.69 (13.85)	0.765
Initial GCS	mean (SD)	6.93 (0.78)	6.93 (0.74)	6.93 (0.76)	0.961
APACHE IV score	mean (SD)	43.09 (2.69)	42.75 (2.53)	42.92 (2.61)	0.607
SOFA score	mean (SD)	9.21(1.34)	9.09 (1.07)	9.15 (1.20)	0.686
Diagnosis *N* (%)	EDH	3 (4.5)	4 (6.1)	7 (10.6)	0.604
	SDH	7 (10.6)	8 (12.1)	15 (22.7)	
	ICH	7 (10.6)	11 (16.7)	18 (27.3)	
	IVH	4 (6.1)	2 (3)	6 (9.1)	
	SAH	4 (6.1)	1 (1.5)	5 (7.6)	
	DAI	8 (12.1)	7 (10.6)	15 (22.7)	
Cause of brain injury *N* (%)	Accident	20 (30.3)	24 (36.4)	44 (66.7)	0.527
	Fall	4 (6.1)	2 (3)	6 (9.1)	
	Internal problems	9 (13.6)	7 (10.6%)	16 (24.2)	
Gender *N* (%)	Male	26 (39.4)	23 (34.8)	49 (74.2)	0.398
	Female	7 (10.6)	10 (15.2)	17 (25.8)	

^*a*^Values are expressed as mean (SD) and frequency (Percentage).

SD, standard deviation; EDH, Epidural Hematoma; ICH, Intracerebral hemorrhage; SAH, Subarachnoid Hemorrhage; ICU, Intensive Care Unit; SDH, Subdural Hematoma; IVH, Intraventricular Hemorrhage; DAI, Diffuse axonal injury; APACHE IV, Acute Physiology and Chronic Health Evaluation version IV; SOFA, Sequential Organ Failure Assessment.

In terms of the family member who performed the sensory stimulation program for the patient in the intervention group, 10 (15.2%) were fathers, 2 (3%) were mothers, 7 (10.6%) were brothers, 3 (4.5%) were sisters, 7 (10.6%) were children, and 4 (6.1%) were spouses of the patients. The mean age of them was 48.12 ± 8.21 and 22 (66.66%) of them were male and 11 (33.34%) female.

To compare the mean differences of GCS and BPS scores before and after each intervention in the study groups, the independent *t*-test was used. The results of this test showed that there is a significant difference between the study groups in terms of mean difference in GCS score before and after the intervention (*P* = 0.001), from the second to the seventh intervention. Also, in terms of the BPS score, there was a statistically significant difference between the two groups in all seven interventions (*P* = 0.001) (Table 2).

**Table 2:** Comparison of study groups in terms of mean difference of GCS and BPS scores before and after each intervention (*N* = 66).

**Variable**	**Intervention number**	**Group**		***t***	**DF**	***P*-value**
		**Intervention** **Mean (SD)**	**Control Mean (SD)**			
GCS	First	0.09(0.29)	0.03(0.17)	1.024	64	0.310
	Second	0.75(0.70)	0.06(0.24)	5.34	64	0.001
	Third	0.81(0.80)	−0.09(0.38)	5.83	64	0.001
	Fourth	1(0.76)	0.12(0.33)	5.44	64	0.001
	Fifth	1 (0.66)	−0.15(0.44)	8.31	64	0.001
	Sixth	1.09(0.57)	0(0.25)	9.93	64	0.001
	Seventh	1.30(0.63)	0.06(0.24)	10.47	64	0.001
BPS	First	−2.03(1.35)	−0.03(0.46)	−8	64	0.001
	Second	−1.93(1.65)	−0.18(0.58)	−5.74	64	0.001
	Third	−1.84(0.72)	0.06(0.65)	−6.08	64	0.001
	Fourth	−2.09(1.52)	0.03(0.30)	−7.82	64	0.001
	Fifth	−2.15(1.30)	−0.06(0.42)	−8.76	64	0.001
	Sixth	−2.54(1.50)	−0.03(0.39)	−9.30	64	0.001
	Seventh	−1.96(2.17)	−0(35)	−5.14	64	0.001

SD, Standard deviation; DF, Degrees of freedom; t, t- statistic.

To examine the changes of the GCS and BPS scores during consecutive measurement times (time effect), across the study groups over the time (group effect), and GCS and BPS score changes over time with respect to grouping (interaction between time and group), the repeated measure ANOVA was used. The results of Mauchly's Sphericity test showed that the correlation coefficients of the consecutive measurements were significantly different (*P* < 0.05). Hence, the correlation equation precondition was not accepted. Therefore, Greenhouse-Geisser correction coefficient was used to report *P*-values.

**Figure 2:**
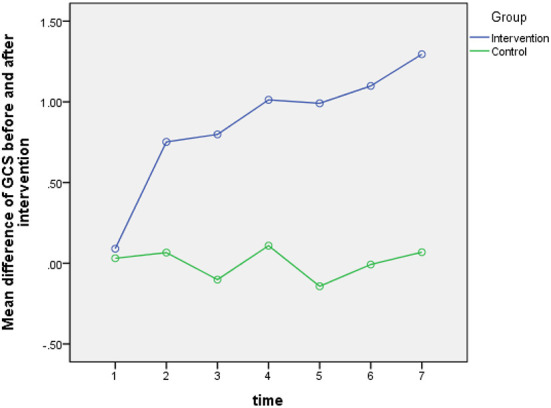
The GCS score changes in the study groups in seven time measurements.

According to the results of Greenhouse-Geisser test for GCS score (the within group comparison), the overall effect of time was not statistically significant (*P* = 0.555). This means that the effect of the intervention on patients' GCS score remained the same during different days. However, the relationship between time and group was statistically significant (*P* = 0.001). While the mean GCS was almost constant during the 7 days in the control group, the mean GCS in the intervention group had almost an increasing trend during these 7 days. These details are given in Table 3 and Figure 2. Moreover, the results of analysis covariance test (the intergroup comparison) showed that the overall effect of the intervention in the experimental group was statistically significant (*p* = 0.001). This means that there is a statistically significant difference between the two groups in terms of their mean GCS scores before and after intervention (*P* = 0.001). The post intervention mean GCS score in the intervention group was higher than that in the control group (Table 4).

**Table 3:** Analysis of within-group effects for GCS and BPS score across the study groups at seven measurement intervals.

**Variable**	**Effects**	**DF**	***f***	***P*-value**
GCS	Overall effect of the intervention Time	5	0.772	0.555
	Time * Group	5	9.02	0.001
	Time * Initial GCS	5	0.216	0.940
	Time * Age	5	0.899	0.472
	Time * APACHE	5	0.513	0.742
	Time * SOFA	5	2.22	0.06
BPS	Overall effect of the intervention Time	5	0.954	0.444
	Time * Group	5	0.974	0.430
	Time * Initial GCS	5	1.594	0.165
	Time * Age	5	0.675	0.635
	Time * APACHE	5	0.802	0.544
	Time * SOFA	5	1.877	0.102

DF, Degrees of freedom; f, f- statistic. *Shows the interaction effect between two variables.

**Table 4:** Analysis of between-group effects for GCS and BPS score across the study groups.

**Variable**	**Effects**	**DF**	***f***	***P*-value**
GCS	Overall effect of Group	1	434.55	0.001
	Overall effect of Initial GCS	1	0.601	0.441
	Overall effect of Age	1	0.057	0.813
	Overall effect of APACHE	1	0.747	0.391
	Overall effect of SOFA	1	0.058	0.810
BPS	Overall effect of Group	1	132.502	0.001
	Overall effect of Initial GCS	1	1.568	0.215
	Overall effect of age	1	0.551	0.461
	Overall effect of APACHE	1	0.515	0.476
	Overall effect of SOFA	1	0.230	0.633

DF, Degrees of freedom; f, f-statistic.

**Figure 3:**
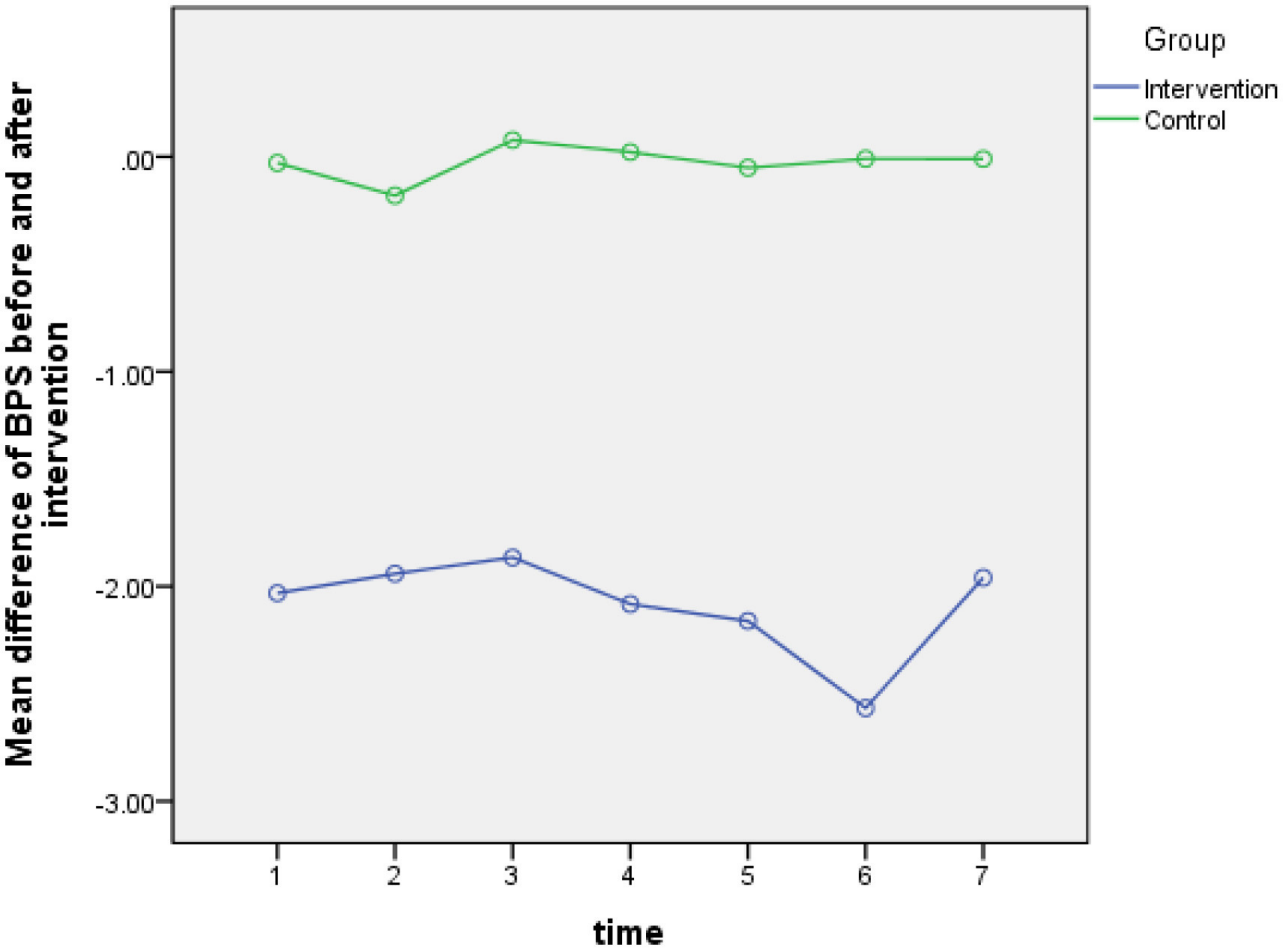
The BPS score changes in the study groups in seen time measurements.

According to the results of Greenhouse-Geisser test for BPS score, the overall effect of time (*P* = 0.444) and the relationship between time and group (*P* = 0.430) was not statistically significant. This means that the effect of the intervention on patients' BPS score remained the same in different days, and no difference was found between the two groups in this regard. These details are given in Table 3 and Figure 3. The results of analysis covariance test (the intergroup comparison) showed that the overall effect of the intervention in the experimental groups was statistically significant (*p* = 0.001). This means that there is a statistically significant difference between the two groups in terms of the mean difference of BPS score before and after intervention (*P* = 0.001). The post intervention mean BPS score in the intervention group was higher than that in the control group (Table 4).

### Qualitative findings

In the qualitative phase of the study, 12 patients in the intervention group who were discharged from the ICU were interviewed. They included eight males and four females, with a mean age of 35.58 years and an average ICU stay of 14.16 days. Quantitative results were complemented with the main themes extracted from the qualitative data after analyzing the patients' interviews. One patient (a 63-year-old woman) did not remember anything special about her ICU stay. Still, the other 11 patients remembered the presence of their family members and receiving sensory stimuli from them. This was under the theme of “A window from limbo to heaven,” as it was the most pleasurable experience, they had during their ICU stay. Most of the patients stated that despite the difficult conditions and many sufferings they went through during their stay in the ICU, this had many benefits for them. The main themes extracted in this phase of study were “Increased consciousness” and “Pain relief.” Also, analysis of patients' experiences showed that they distinguished between receiving sensory stimulation from family members and the other sensory stimulations they may have received as part of routine care provided by health care personnel.

“*Sometimes the nurses would talk to me…. One of them put his hand on my forehead and told me: Don't worry, you will be fine…… he would then bend and extend my arms and legs…. this is very good…. But I waited impatiently to hear my son's voice again…. It was different from everything else…. My troubled mind was calmed down just by hearing my son's voice and touching his hands.” (40-year-old man)*

The results obtained in this phase of study are in line with the results of the quantitative phase. By examining the patients' experiences, we can understand why and how the intervention could cause a difference in the level of consciousness and pain intensity between the intervention and control groups. Table 5 presents the main themes and illustrative quotes for each theme.

**Table 5:** Illustrative quotes (The presence of family: “A Window from Limbo to Heaven”).

**Theme**	**Quotation**
Increased consciousness (11 patients)	“*Whenever I heard my daughter's voice, it was like a spark in my brain, removing all my dizziness. Whenever she came, I had a deeper feeling of her presence. She was exercising my arms and legs. She helped me open my eyes and showed me old pics. I can remember these much better” (41-year-old woman) “Farzad! (the patient's name) …. Open your eyes!… I have come to see you, my son!…” As soon as I heard my mom's voice and felt her hands' touch, I, who was dizzy and confused until that moment, got oriented and mindful. The only thing I liked to hear was this voice, not all the clatter of the devices and the staff” (27- year- old man)*
Pain relief (11 patients)	“*He (the patient's brother) would massage my arms and legs, bending and extending them, and I felt the fresh blood circulating all over my body…and it would relieve my pain like pouring water over fire” (25- year- old man). “Amidst the turmoil of the pain and suffering I was through, he (the patient's son) would come here and massage my arms and legs and caress my face; he even played my favorite songs, which was better than a hundred painkillers” (40-year-old man)*
A sense of relaxation (11 patients)	“*My stress and anxiety was replaced by a lovely serenity as I heard his (the patient's father) voice, when I listened to relaxing music, or when I inhaled the scent of narcissus flowers he would bring me every day. It was like being floating in a pool of water after a hard day's work” (34-year-old man) “Her (the patient's wife) presence was like a window from limbo to heaven. In her presence, all my stress and anxiety turned into a pleasant serenity and I was freed from all that confusion” (42 years old)*
Sense of security and confidence (10 patients)	“*He (the patient's father) made me feel more secure. His presence was encouraging, and I was sure whatever was going to happen, he would be there by my side. Although I couldn't utter a word, he would know what I meant and what I asked for.” (34-year-old man) “Now, I had someone (the patient's mother) who cared for me as always… It was a good sense of confidence despite all the stress and worries… I loved to act coyly as I was sick at home, and he would made a real fuss of me” (26-year-old woman)*
Increased energy and motivation to get back to life (10 patients)	“*He (the patient's father) talked about what was going on at home; he told me everyone was waiting for me to come back… I heard my mother's voice: “Ali!… My son! You will be well soon… We are all waiting for you to come home…” It was like watering a withered flower… I felt my energy and ability for getting better increased tenfold in those moments.” (29-year-old man) “Hearing my daughter's voice under those circumstances was like a bomb of energy for me. When she was there, I could be more courageous than I used to be. This made me feel stronger and think I can get back to life… I was trying to breathe more slowly to get rid of this tube (endotracheal tube) as soon as possible.” (41-year-old woman)*
Feeling the flow of life (9 patients)	“*When my father was beside me, I could think of good things amidst all the stress and bad thoughts I had. For example, I even thought of continuing my education and I wanted to get my PhD…You know… in those moments, I felt that life was still flowing on” (29-year-old man) “Imagine how comforting it was to hear your favorite music in that limbo. Yes…there was still life outside of this limbo. At that time, I thought that the whole world was summarized in the ICU” (27-year-old woman)*
Hardships becoming easier to tolerate (7 patients)	“*When she (patient's wife) came, by hearing her voice and touching her hands, I was more tolerant of everything… all those tubes and devices connected to me…; that room was like solitary confinement” (42- year-old man). “I do not know why, but the situation was much better when she (the patient's mother) was with me, and she was talking to me, and I could feel the warmth of her hands… Everything seemed to be easier… Staying in that little bed was painful. I could breathe more easily, and my heart beating more calmly.” (26-year-old woman)*
Feeling of being important to family members (4 patients)	“*My father came to visit me every day… I felt that I was irreplaceable in my family, which was the best thing I could feel” (34- year-old man). “When Vahid (the patient's brother) came to me, he made a phone call to home, and I heard their voices… They were saying that they were all waiting for me to return home. They had even prepared my room. I did not think that I had been so valuable to my family” (25-year- old man)*
Overcoming the fear of death (4 patients)	“*When my daughter was here, and I listened to the Quran, death was no longer scary for me… You know, even if I was going to die, I would die beside my daughter” (41-year-old woman) “When she (the patient's wife) touched my hands and played the voice of my children, my fear and anxiety of dying diminished… I was afraid to die and never see my family again” (42-year-old man)*.

## Discussion

The results of the present study showed a significant difference between the intervention and control groups in terms of the patients' mean consciousness score, which was higher in the intervention group compared with the control group. Unlike patients in the control group, patients in the intervention group experienced an upward trend in their consciousness levels from the second day after the intervention. Most of these patients also mentioned that receiving SSP from family members during their stay at the ICU promoted their consciousness, improved their sense of time and place, and reduced their dizziness and confusion. This result can be explained from the perspective of neuroscience: Familiar sensory stimulations activate the limbic system, which increases the sympathetic system's activity. As a result, the norepinephrine level in nerve terminals is elevated, and messages are transmitted faster and better to the cerebral cortex and are interpreted at the center of its emotional response which this leads to increased consciousness and arousal of the patient (14). Balanced sensory stimulation can also cause changes in healthy nerve fibers in the brain (hypertrophy and budding of new synapses), which can help reorganize brain activity and synaptic nerves and activate the RAS system and the cerebral cortex (30). The present study showed that receiving SSP from family members can have a special and tangible effect. Consistent with the results of the present study, Adinehvand et al. (10) and Salmani et al. (14), in their studies showed that the consciousness level of brain injury patients admitted to the ICU who received sensory and emotional stimulation programs by family members was higher compared with the group receiving these stimulations by nurses or other healthcare personnel.

Examining the experiences of patients in the intervention group in the present study can help clarify the reason for this. Most patients distinguished between the impact of sensory stimulation they received from family members and that other sensory stimulation they received from healthcare personnel as part of routine care. As the patients themselves stated, in the difficult and exhausting conditions of the ICU, the presence of a family member and receiving familiar sensory stimuli such as hearing a family member's voice and feeling their presence was something that the patients loved and eagerly looked forward to. Normally, people take refuge in the open arms of their family members during times of hardships and troubles. Staying in the ICU is a case in point which could be made easier by the presence of a family member and receiving sensory stimuli from them. Moreover, by receiving this form of stimulation, the patients' minds will be calmed down and free from confusion and anxiety, making them feel hopeful, energetic, and safe. This can contribute to the improved functioning of the central nervous system and promotion of their consciousness and alertness. However, for the patients of the control group, the SSP was not implemented by family members, which could explain their relatively lower consciousness scores. According to Gomez et al.'s meta-synthesis (31), one of the most important negative realities perceived by ICU patients is unfamiliar sensory stimulations such as constant noise made by devices and staff and being separated and distant from family members and acquaintances.

As far as pain intensity was concerned, our study results showed a significant difference between the mean pain score of patients in the intervention and control groups, with the mean pain score of patients in the intervention group being lower than that in the control group. However, pain in these patients did not have a downward trend in different days. This could be due to the nature of pain because even the effect of painkillers on pain has a certain time limit (32). Most of the interviewed patients also stated that the family member's presence and receiving SSP from them reduced their pain in various ways. The most important items cited include: the emotional support by family members, forgetting the pain when a family member was present and while listening to music, and massaging and moving the limbs and joints by a family member and making pain more tolerable. A mixed-method by study Ames et al. (33) showed that listening to favorite music postoperatively reduced pain in patients admitted to the ICU. After discharge from the ICU, the patients stated that listening to music in the ICU had positive effects such as reducing stress and anxiety and causing them to forget the pain. In another study by Tronstad et al. (34), patients reported reduced pain when they received familiar sensory stimulations such as hearing familiar sounds and touching family members' hands. Khoja et al.'s study (16), showed that auditory stimulation with family members' voices reduces the pain intensity of patients admitted to the ICU (16). Jagan et al. (9) also concluded that massage and touch therapy interventions could increase consciousness levels and reduce pain in patients admitted to the ICU.

## Limitations

First, despite the researchers' insistence that family members should visit the patient for more than 1 h a day, due to the restrictions caused by the COVID-19 disease, this permission was not granted by the organization responsible for the study site. The second limitation is that our study compared the effects of stimulation by family members with no stimulation. Probably a condition of stimulation by neutral/unfamiliar people could have been a better control condition. However, the results of the qualitative phase showed that patients distinguished between receiving SSP from the family members and other sensory stimuli as part of routine care they received from unfamiliar people (healthcare personnel). The final limitation is that this study did not evaluate long-term consequences that can be caused by the intervention, which include reducing the patients' functional and cognitive impairments after ICU discharge and improving their quality of life. Meanwhile, many critically ill patients have been reported to suffer from long-term cognitive impairment due to ICU admission (4, 35), which affects their quality of life and the effectiveness of intensive care (36, 37). Long-term cognitive effects are related to the stressful situations that patients experience during their stay in the ICU (38), and receiving SSP by family members can be a source of relaxation and reduce patient stress. Therefore, it is recommended that these factors be further explored in future studies. In addition, considering that in the present study, the family member who performed the sensory stimulation program for the patient was the same during the study, for further investigation, it is suggested that in the next studies, this person should be selected from the family members in rotation.

## Conclusion

Comparing and combining the results of the quantitative and qualitative phases of the present study showed that despite the many hardships and sufferings that brain injury patients experience during their ICU stay, meeting with a family members and receiving sensory stimulation from them has many positive outcomes such as increasing consciousness and reducing pain in these patients. These positive results are in line with the results of quantitative studies that have been conducted in relation to this subject and those of the present study. Despite this evidence, SSP by family members is not still implemented as part of standard care for patients in many ICUs. Therefore, it is necessary for health policymakers and ICU healthcare personnel to provide the framework and facilities to benefit from this willing free workforce (i.e., patient's family) who are always present agitatedly behind the ICU doors for the better care of their patients.

## Data availability statement

The original contributions presented in the study are included in the article/supplementary material, further inquiries can be directed to the corresponding author.

## Ethics statement

This study involving human participants that were reviewed and approved by the Ethics Committee of Ahvaz Jundishapur University of Medical Sciences (Ref. ID: IR.AJUMS.REC.1400.014) and the Iranian Registry of Clinical Trials (Ref. ID: IRCT20120414009469N4). Ethical considerations were in accordance with the Declaration of Helsinki 1995, revised in 2001. The aim and method of the study were explained to the family members of the patients, and the first author answered their questions. Family members and their patients could withdraw from the study at any time without any effect on the caring process of the patient. A written informed consent form was signed by a family member (patient's legal guardian) who willingly agreed to take part in this study. The confidentiality and anonymity of patient information were ensured throughout the study process.

## Author contributions

Study conception, design, and critical revision of the manuscript: MA and NE. Data collection: MA, SM, and MS. Data analysis and interpretation: MA, NE, and SJ. Drafting of the manuscript: MA, NE, SM, SJ, and MS. All authors contributed to the article and approved the submitted version.

## Funding

This work was supported by the Research Deputy of Ahvaz Jundishapur University of Medical Sciences (Grant Number U-00010).

## Conflict of interest

The authors declare that the research was conducted in the absence of any commercial or financial relationships that could be construed as a potential conflict of interest.

## Publisher's note

All claims expressed in this article are solely those of the authors and do not necessarily represent those of their affiliated organizations, or those of the publisher, the editors and the reviewers. Any product that may be evaluated in this article, or claim that may be made by its manufacturer, is not guaranteed or endorsed by the publisher.
